# Abnormal intrinsic functional hubs and connectivity in stable patients with COPD: a resting-state MRI study

**DOI:** 10.1007/s11682-019-00130-7

**Published:** 2019-06-11

**Authors:** Haijun Li, Huizhen Xin, Jingjing Yu, Honghui Yu, Juan Zhang, Wenjing Wang, Dechang Peng

**Affiliations:** 1 Department of Radiology, the First Affiliated Hospital of Nanchang University, No.17, Yongwai Zheng Street, Donghu District, Nanchang, 330006 Jiangxi Province People’s Republic of China; 2 Department of Respiratory, the First Affiliated Hospital of Nanchang University, Nanchang, China

**Keywords:** Chronic obstructive pulmonary disease (COPD), Resting state fMRI, Degree centrality, Functional connectivity, Cognitive impairment

## Abstract

Chronic obstructive pulmonary disease (COPD) affects a large population and is closely associated with cognitive impairment. However, the mechanisms of cognitive impairment in COPD patients have not been unraveled. This study investigated the change in patterns of intrinsic functional hubs using a degree centrality (DC) analysis. The connectivity between these abnormal hubs with the remaining brain was also investigated using functional connectivity (FC). Nineteen stable patients with COPD and 20 normal controls(NC) underwent functional magnetic resonance imaging (MRI) examinations and clinical and neuropsychologic assessments. We measured the voxel-wise DC across the whole brain gray matter and the seed-based FC between these abnormal hubs in the remaining brain matter; the group difference was calculated. A partial correlation analysis was performed to assess the relationship between the abnormal DC and clinical variables in COPD patients. Compared to NC, the patients with COPD exhibited significantly decreased DC in the right lingual gyrus (LG), bilateral supplementary motor area (SMA), and right paracentral lobule (PCL). A further seed-based FC analysis found that COPD patients demonstrated significantly decreased FC between these abnormal hubs in several brain areas, including the left cerebellum anterior lobe, left lingual gyrus, left fusiform gyrus, right insula, right inferior frontal gyrus, limbic lobe, cingulate gyrus, left putamen, lentiform nucleus, right precuneus, and right paracentral lobule. A partial correlation analysis showed that the decreased DC in the right PCL was positively correlated with the FEV1 and FEV1/FVC, and the decreased DC in the SMA was positively correlated with naming and pH in COPD patients. This study demonstrates that there are intrinsic functional hubs and connectivity alterations that may reflect the aberrant information communication in the brain of COPD patients. These findings may help provide new insight for understanding the mechanisms of COPD-related cognitive impairment from whole brain functional connections.

**Electronic supplementary material:**

The online version of this article (10.1007/s11682-019-00130-7) contains supplementary material, which is available to authorized users.

## Introduction

Chronic obstructive pulmonary disease (COPD) is a major cause of death that is characterized by persistent and progressive airflow limitation associated with an abnormal inflammatory response of the lungs to noxious particles or gases.(Vogelmeier et al. 2017) According to the World Health Organization, COPD is currently the fourth leading cause of death worldwide, and by 2030, it is predicted to be the third leading cause of death. The primary clinical manifestations of COPD is airflow obstruction, which is associated with many comorbidities and leads to increased morbidity, reduced quality of life, and increased mortality.(Watz et al. 2008) Neuronal damage and cognitive dysfunction is one of the most important comorbidities of COPD.(Meek et al. 2001; Cleutjens et al. 2014; Dodd et al. 2010; Baird et al. 2017) A systematic review reported that 25% of COPD patients had mild cognitive impairment, and 32% of COPD patients had any cognitive impairment.(Yohannes et al. 2017) In addition, cognitive impairment may increase shortness of breath and fatigue, leading to the incorrect use of inhaler devices; this may increase the risk of deterioration and result in serious health consequences.(Dodd et al. 2013) The main pathophysiological mechanism of cognitive dysfunction in COPD, which includes oxidative stress and hypoxia/hypercarbia, is not yet fully understood.(Wouters et al. 2002) The brain is extremely sensitive to changes in arterial oxygen concentration; therefore, brain pathology is a potential systemic manifestation that may explain the increasingly reported cognitive impairment in COPD.

Many previous studies have confirmed that patients with COPD display not only structural changes but also metabolic abnormalities in local functions; these changes can be observed in specific brain regions using advanced neuroimaging techniques. These changes include diminutions gray matter volume and gray matter density, including the frontal cortex, cingulate cortex, anterior insula, and hippocampal atrophy, which can partly explain the pathophysiological and psychological changes in COPD patients.(Zhang et al. 2013; Li and Fei 2013; Cleutjens et al. 2017) An amplitude of low-frequency fluctuation(ALFF) study found that COPD patients showed significant decreases in ALFF values in the bilateral posterior cingulate gyri and right lingual gyrus, which may have a pathophysiological meaning.(Zhang et al. 2016) A single-photon emission computed tomography study showed significantly decreased brain perfusion in the frontal and parietal lobes of COPD patients, which was correlated with neuropsychological tests.(Ortapamuk and Naldoken 2006) A magnetic resonance spectroscopy study found significantly lower N-acetyl aspartate (NAA), creatine (Cr) and choline (Cho) levels in parietal white matter, which was positively correlated with memory dysfunction in severe COPD patients.(Shim et al. 2001) A functional connectivity (FC) study reported that COPD patients showed an abnormal default network connection, which may be related to cognitive impairment.(Hu et al. 2018) A multimodal MRI study found widespread reduced white matter integrity and increased gray matter activation in resting-state networks, which appeared to account for the difference in cognitive performance.(Dodd et al. 2012) These studies have focused on local spontaneous brain activity or have analyzed the FC or network within the selected brain regions based on a priori assumption. However, the human brain is a complex network with nodes representing brain regions that contain neurons and edges representing neural connections; this network is thought to chart the circuits that underlie the physiological basis for information processing and mental representations.(Bullmore and Sporns 2009) However, until now, no study has investigated intrinsic function hubs and the connectivity between these abnormal hubs with the remaining brain and the pathogenesis of cognitive dysfunction in COPD.

The voxel-wise degree centrality (DC), which is a network measurement method based on graph theory with nodes and edges, can assess the whole brain functional connectome without requiring a priori nodes or a region of interest (ROI). When a given voxel or node has many connections to other voxels or nodes, it is considered a high DC (hub).(Zuo et al. 2012) Unlike independent component analysis or seed-based FC, the voxel-based DC measurement directly and quantifiably reflects the functional relationship changes of a given voxel or node in the whole brain functional network connectome. Previous studies have also confirmed that DC has a high sensitivity, specificity, and test-retest reliability,(Zuo and Xing 2014) and it has been increasingly used in many disorders associated with cognitive impairment, such as obstructive sleep apnea, diabetes, Parkinson’s, and Alzheimer’s disease.(Li et al. 2016; Liu et al. 2018; Wang et al. 2018; Guo et al. 2016) Therefore, the voxel-wise DC can help us investigate the intrinsic function hubs that may be associated with cognitive impairment in COPD.

In the present study, we hypothesized that abnormal intrinsic function hubs and their connectivity may contribute to cognitive deficits in COPD patients. First, the mean voxel-wise DC was computed to identify the intrinsic functional hubs in two groups and to investigate the potential altered DC in stable patients with COPD compared to normal controls (NC). Next, the seed-based FC approach further to evaluated the connectivity abnormality by placing the ROI in the clusters that exhibited significantly altered DC in patients with COPD. We also investigated the relationships of brain functional hubs alterations with clinical data and neuropsychological performance in COPD patients.

## Materials and methods

### Subjects

This study recruited 19 stable patients with COPD and 20 age- and education-matched NC from the Respiratory Department of the First Affiliated Hospital of Nanchang University (Nanchang, China) from December 2017 to June 2018. All participants underwent a detailed clinical history and physical examination. Moreover, they underwent a lung function test to ensure that all patients were in a stable state (with no exacerbations during the past 8 weeks) or were in a stable state after therapy. The diagnoses and classifications of COPD were based on the Global Initiative for Chronic Obstructive Lung Disease (GOLD) guidelines from 2017.(Vogelmeier et al. 2017) The exclusion criteria were as follows: 1) obstructive sleep apnea syndrome or insomnia; 2) neurological and psychotic disorders, such as dementia, psychosis, epilepsy and etc.; 3) cerebral tumor or trauma or stroke or postcraniotomy; 4)heart failure; 5) history of drugs and/or alcoholism; 6) comorbidities such as diabetes, anemia, liver failure and cardiovascular disease; 7) the Montreal Cognitive Assessment (MoCA) and the Mini-mental State Examination (MMSE) evaluations could not be completed; and 8) MRI contraindications, such as claustrophobia, metallic implants, or devices in the body.

### Pulmonary function

The forced expiratory volume in 1 s (FEV_1_), postbronchodilator FEV_1_/forced expiratory vital capacity (FVC) and forced vital capacity (FVC) were measured. According to the GOLD manual, the subjects with a FEV_1_/FVC < 0.7 and FEV_1_ ≥ 80% predicted were classified as mild COPD, those with 50% ≤ FEV_1_ < 80% predicted were classified as moderate COPD, those with 30 ≤ FEV_1_ < 50% predicted were classified as severe COPD and those with FEV_1_ < 30% predicted were classified as extremely severe COPD. These data were obtained from the mouth spirometer device (Erich Jaeger GmbH, Hoechberg, Germany) 15 min after the patients inhaled 400 μg of salbutamol (Ventolin; GlaxoSmithKline, London, UK).

### Arterial blood gas analysis

The arterial blood was measured using Stat Profile Critical Care Xpress (Nova Biomedical, Waltham, MA, USA) for 30 min. The following indicators were recorded: power of hydrogen (pH), blood oxygen saturation (SaO_2_), arterial partial pressure of oxygen (PaO_2_), and arterial partial pressure of carbon dioxide (PaCO_2_).

### Cognitive assessments

Cognitive function was assessed using MoCA and MMSE. The MoCA contains eight different cognitive fields, including visuospatial and executive function, naming, memory, attention, language, abstraction, and orientation. The total MoCA score is 30. A total MoCA score greater than or equal to 26 indicates normal functioning, whereas a score less than 26 reveals cognitive impairment. If the subject has had less than 12 years of education, the total score increases by one point to adjust for educational bias.(Nasreddine et al. 2005) The MMSE is one of the most influential standardized intelligence state inspection tools. A total MMSE score between 27 and 30 is normal. A score of less than 27 indicates cognitive impairement.(Folstein et al. 1975) All participants were asked to complete the two cognitive scale assessments guided by two independent neuropsychologists.

### MRI data acquisition

All the MRI datas were collected on 3.0 Tesla MR scanners (Siemens, Erlangen, Germany) with 8-channel head coils. All participants laid down on the scanning bed; earplugs were used to minimize scanner noise, and foam pads were used to reduce head movements. The participants were asked to stop thinking as soon as possible and to closed their eyes, but remain awake during MRI scan. First, conventional T2-weighted images (repetition time (TR) = 4000 ms, echo time (TE) = 113 ms, slices = 19, 5-mm slice thickness, 1.5-mm gap, field of view (FOV) = 220 mm × 220 mm) and T1-weighted images (TR = 250 ms, TE = 2.46, the same slices, slice thickness, gap, and FOV to T2WI) and were acquired. A total of 176 high-resolution T1-weighted images were collected using sagittal orientation three-dimensional spoiled gradient-recalled echo sequence (TR = 1900 ms; TE = 2.26 ms; 1.0-mm thickness with no gap; acquisition matrix = 256 × 256; FOV = 250 mm × 250 mm, flip angle = 9°); 30 axial slices and 240 functional images were obtained using gradient-recalled echo-planar imaging pulse sequence (TR = 2000 ms; TE = 30 ms; 4.0-mm thickness; 1.2-mm gap; acquisition matrix = 64 × 64; FOV =230 mm × 230 mm; flip angle =90°).

### Functional MRI data preprocessing

Before preprocessing, the functional images were checked using MRIcro software (www.MRIcro.com), and the T1WI and T2WI images were reviewed by 2 senior radiologists to rule out potential confounding factors such as general structure damage and lack of data, etc. The preprocessed MRI data were analyzed using Statistical Parametric Mapping (SPM8) (http://www.fil.ion.ucl.ac.uk/spm), and Data Processing & Analysis for Brain Imaging (DPABI) (http://rfmri.org/DPABI) based on the MATLAB2014a (Mathworks, Natick, MA, USA) platform. The first ten volumes of each participants were removed to guarantee the participants’ adaptation to the scanning environment and to allow the signal to reach equilibrium. Then, slice timing and three-dimensional head motion correction were processed for the remaining time series. No participants exceed the maximum cardinal direction displacement (x, y, z) of less than 2.0 mm and maximum spin (x, y, z) of less than 2.0°; frame-wise displacement (FD) was more than 2.5 standard deviations based on the method of (Van Dijk et al. 2012) The high-resolution T1-weighted and functional images were manually realigned to the anterior commissure, and structural images were coregistered to the functional images of each subjects using a linear transformation.(Ashburner and Friston 2005) In sequence, the transformed structural images were segmented into white matter, gray matter, and cerebrospinal fluid using the new segmentation in SPM8. Then, the images were normalized to the standard Montreal Neurological Institute (MNI) template using the Diffeomorphic Anatomical Registration Through Exponentiated Lie Algebra tool and resampled to 3 mm × 3 mm × 3 mm voxels. Friston 24-parameter, white matter signal, and cerebrospinal fluid signal were regressed out from the time series of all voxels using linear regression.(Friston et al. 1996) After filtering with a temporal filter (0.01–0.08 Hz), the resulting images were smoothed using a Gaussian kernel of 6-mm full width at halfmaximum.

### Voxel-wise degree centrality

Based on preprocessing, DC values were computed in the default gray matter mask using DPARSF(http://rfmri.org/DPARSF) according to previous studies.(Li et al. 2016) In brief, for each subject, Pearson’s correlation coefficients were calculated between the time series for any pair of voxels. According to the adjacency matrix of the graph, voxle-wise DC can be calculated according to following equation(Zuo et al. 2012):

1 $$ DC(i)=\sum \limits_{j=1}^N{r}_{ij}\left({r}_{ij}>{r}_0\right)\Big) $$

ih which r_ij_ is the correlation coefficient between voxel i and voxel j and r_0_ is a correlation threshold that is set to eliminate weak correlations.(Zuo et al. 2012; Hou et al. 2014; Yan et al. 2013) To increase the stability and repeatability, five different correlation thresholds (r_0_ = 0.15, 0.2, 0.25, 0.3 and 0.35) were analyed in this study. The voxel-wise DC map for each subject was converted into a z-score map.

### Seed-based functional connectivity analysis

Although a DC analysis can determine whether the brain functional connectivity with other brain regions has changed, it is unable to provide detailed information on the connection between the brain areas and the specific region has changed. Therefore, we used the seed-based FC approach to further investigate the connectivity abnormality by placing the ROI that exhibited significantly altered DC in COPD patients. According to the results, the following ROI were chosen: right LG (x = 21, y = −69 and z = −12), SMA (x = 6, y = −9 and z = 60), and right PCL (x = 2, y = −54 and z = 69). The mean time series of each ROI was extracted, and Pearson’s correlation analysis for each individuals was conducted between the seed ROIs and the remaining voxels within the gray matter mask. The resulting correlation maps were z-transformed for the group level voxel-wise t-test analyses.

### Statistical analysis

Intergroup comparisons of demographic and clinical data andneuropsychological test scores between the COPD patients and NC were assessed using ths IBM Statistical Package for the Social Sciences 19.0 software (IBM SPSS 19.0, Chicago, IL, USA). First, the data distribution was verified using the Kolmogorov-Smirnov test. Second, independent sample t-tests and the Mann-Whitney U test were applied to normally distributed continuous data and to nonnormally distributed data, respectively. The sex proportion was examined with the χ^2^ test. *P* < 0.05 indicated statistical significance.

To identify the spatial pattern of intrinsic functional hubs in the two groups in the DC map, one sample *t*-tests were performed with the base of “0” in the two groups, respectively. To further test intergroup differences in DC, we performed independent two-sample *t*-tests using age, sex and education as nuisance covariates within the gray matter mask using the REST V1.8 software. Smoking is one of the most important factors in COPD pathogenesis, and it may affect intrinsic functional connectivity. An additional analysis was performed using smoking as a nuisance covariates to avoid potential effects. All results were reported at the significant voxel level of *P* < 0.01, the cluster level of *P* < 0.05, and Gaussian random field theory (GRF)-corrected.

For the seed-based FC map, an independent sample t-test was applied to investigate the inter-group differences between COPD patients and NC using age, sex and educational as nuisance covariates within the default gray matter mask using the REST V1.8 software. Results with a voxel level of *P* < 0.01, cluster level of *P* < 0.05, and GRF-corrected were considered statistically significant.

Moreover, to assess the relationship between altered DC brain regions and clinical variables in patients with COPD, each significantly altered DC brain areas was preserved as a mask, and the mean coefficient of connection was extracted from every patient. A partial correlation analysis was performed to assess the relationship between the decreased mean DC values and clinical variables in patients with COPD using IBM SPSS with age, sex and education as nuisance covariates; a statistical significance level of *P* < 0.05, corrected for multiple comparisons, was used.

## Results

### Demographic and clinical data

The demographic and clinical characteristics of the two groups are summarized in Table 1. No significant intergroup differences were found in terms of age, sex, or education. The COPD patients had significantly lower scores for SaO_2_, PaO_2_, FVC, FEV1, FEV1/FVC, MMSE, MoCA, naming, visuospatial and executive function, and memory, and had significantly higher scores for PaCO_2_ and pack-years than the NC. The intracranial volume revealed no significant differences between the two groups.

**Table 1:** Demographic and clinical characteristics of COPD and NC

Characteristic	COPD(*N* = 19)	NC(*N* = 20)	*P* value
age, years	62.7 ± 5.9	60.8 ± 6.3	0.361
men /female,n	14/5	15/5	0.925
education, years	5.5 ± 3.2	6.3 ± 2.7	0.417
Disease duration(years)	4.5 ± 5.6	/	/
Pack-years	27.9 ± 20.5	8.9 ± 6.3	<0.001
SaO_2_(%)	95.5 ± 2.6	97.8 ± 1.8	0.008
PaO_2_ (mm Hg)	82.6 ± 16.5	98 ± 19.5	<0.001
PaCO_2_ (mm Hg)	49.3 ± 8.0	38.5 ± 4.3	<0.001
Respiratory rate,times/min	19.5 ± 0.6	18.5 ± 1.2	0.654
FVC (% predicted)	67.5 ± 19.9	96.5 ± 15.8	<0.001
FEV1 (% predicted)	46.1 ± 20.6	97.1 ± 16.9	<0.001
FEV1/FVC (%)	55.8 ± 16.3	81.2 ± 8.3	<0.001
MMSE	22.4 ± 3.6	27.2 ± 2.2	<0.001
MoCA	18.4 ± 4.3	26.4 ± 3.2	<0.001
Naming	2.6 ± 0.5	2.9 ± 0.2	0.026
Visuospatial and executive function	3.6 ± 1.1	4.3 ± 0.9	0.012
Attention	5.3 ± 1.0	5.8 ± 0.7	0.075
Language	1.4 ± 0.8	1.8 ± 0.6	0.235
Memory	2.6 ± 1.3	3.9 ± 1.2	<0.001
Abstraction	1.2 ± 0.7	1.4 ± 0.6	0.231
Orientation	5.5 ± 0.6	5.9 ± 0.1	0.356
Intracranial volume (cm^3^)	1540.20 ± 110.49	1542.79 ± 99.17	0.895

Abbreviations: *COPD* Chronic obstructive pulmonary desease, *NC* Normal controls, *SaO2* Blood oxygen saturation, *Pao2* Arterial partial pressure of oxygen, arterial *PaCO2* Partial pressure of carbon dioxide, *FEV1* Forced expiratory volume in 1 s, *FVC* Forced expiratory vital capacity, *MoCA* Montreal cognitive assessment, *MMSE* The Mini-mental state examination

### Weighted degree centrality differences between groups

**Fig. 1:**
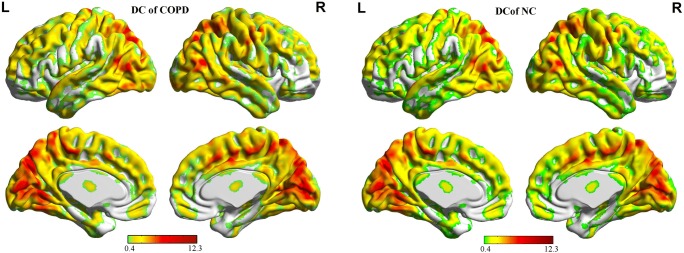
Highly similar spatial distributions of the higher DC value(functional hubs) in the COPD group and NC group(r_0_ = 0.25)

**Fig. 2:**
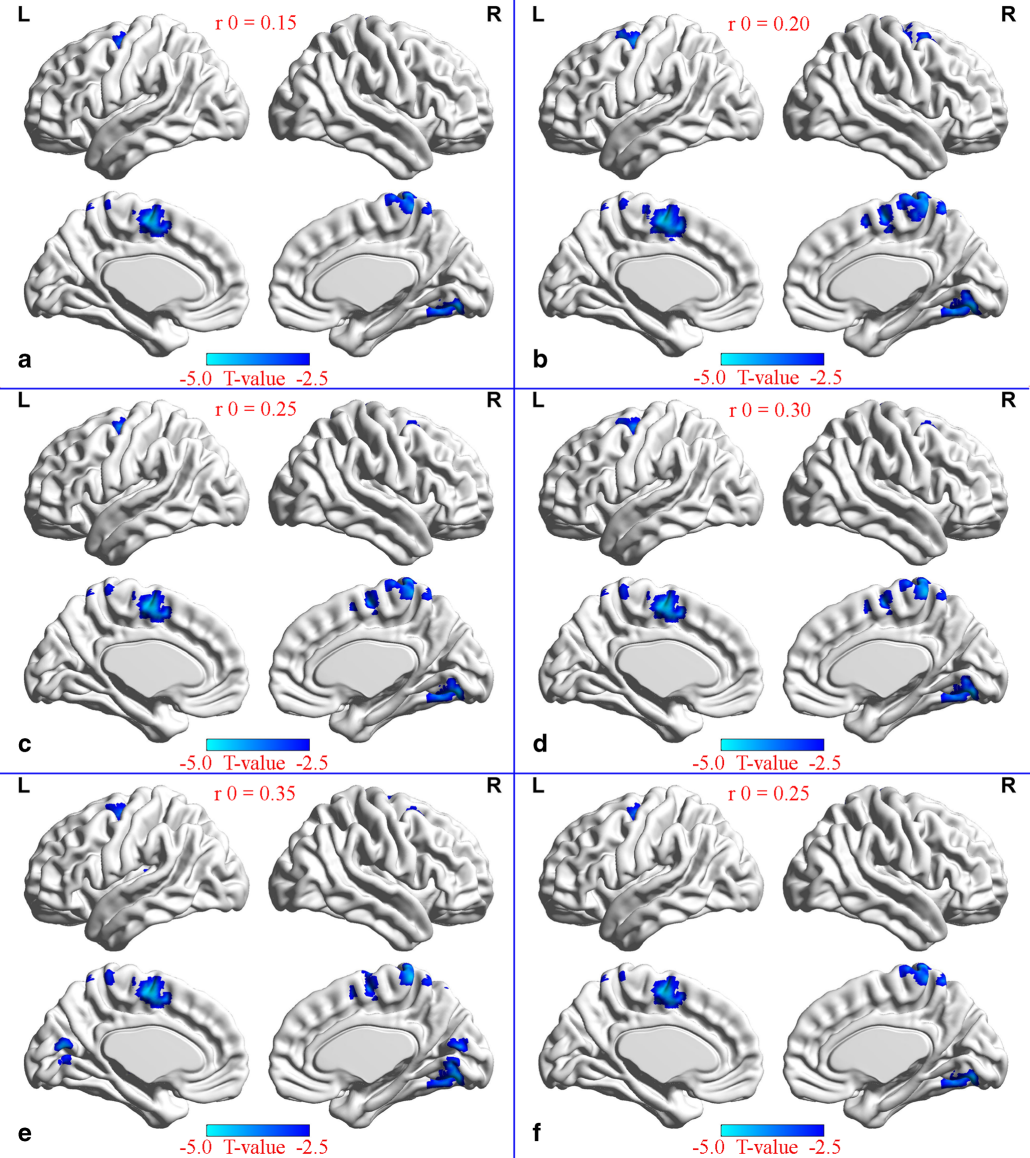
The significantly altered DC map in COPD patients were remarkably similar in different correlation thresholds(r_0_ = 0.15, 0.2, 0.25, 0.3 and 0.35) (**a**~**e**), and after controlling for smoking (r_0_ = 0.25) (**f**) compared to NC

**Fig. 3:**
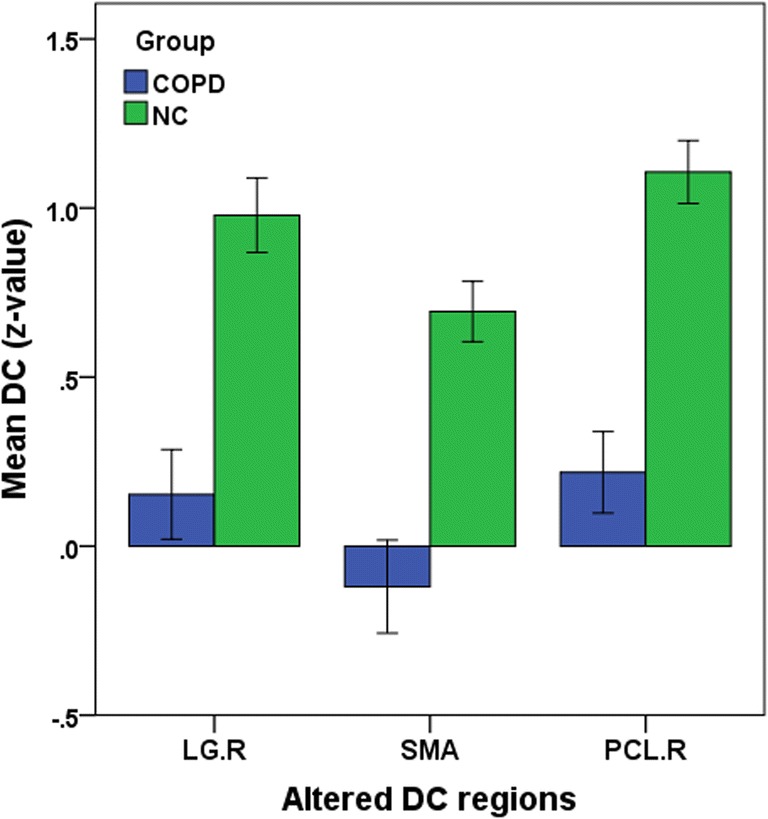
Mean weighted DC signal values for altered regional brain areas between COPD patients and NC. Abbreviations: *DC* Degree centrality, *COPD* Chronic obstructive pulmonary desease, *NC* Normal controls, *LG* Lingual gyrus, *SMA* Supplementary motor area, *PCL* Paracentral lobule, *R* Right

Compared to the global mean value, both groups displayed similar higher DC values (functional hubs) in the posterior cingulate cortex, cuneus, lingual gyrus, occipital cortex, superior frontal cortex, supplementary motor area, and paracentral lobule (Fig. 1), and the intergroup differences were also remarkably similar at different correlation thresholds(r_0_ = 0.15, 0.2, 0.25, 0.3 and 0.35) (Fig. 2a-e). These results were similar to the significant clusters after controlling for smoking, with the exception of the absence of the left supplementary motor area (SMA) clusters (Fig. 2f). Therefore, we primarily report the results based on the weighted graph of positive correlations (r_0_ = 0.25). Compared to NC, the patients with COPD showed a significantly reduced DC in the right lingual gyrus (LG), bilateral SMA, and right paracentral lobule (PCL), but no increased DC was found (Table 2, Fig. 2). Meanwhile, the average DC z-values of the altered brain regions wra shown in Fig. 3.

**Table 2:** Brain regions showed reduced DC between the COPD and NC(r_0_ = 0.25)

Condition	Brain regions	BA	Peak MNI			t-value	Cluster (voxels)
			X	Y	Z		
COPD<NC	Lingual Gyrus.R	18	21	−69	−12	−5.1025	111
COPD<NC	Supplementary Motor Area	6	−9	−3	60	−5.2272	154
COPD<NC	Paracentral Lobule.R	4	2	−54	69	−4.3777	74

Abbreviations: *COPD* Chronic obstructive pulmonary desease, *NC* Normal controls, *MNI* Montreal neurological institute, *BA* Broadmann area, *R* Right, *L* Left, *GRF*-Corrected, voxle level *P*<0.01, cluster level *P*<0.05

### Seed-based functional connectivity

**Fig. 4:**
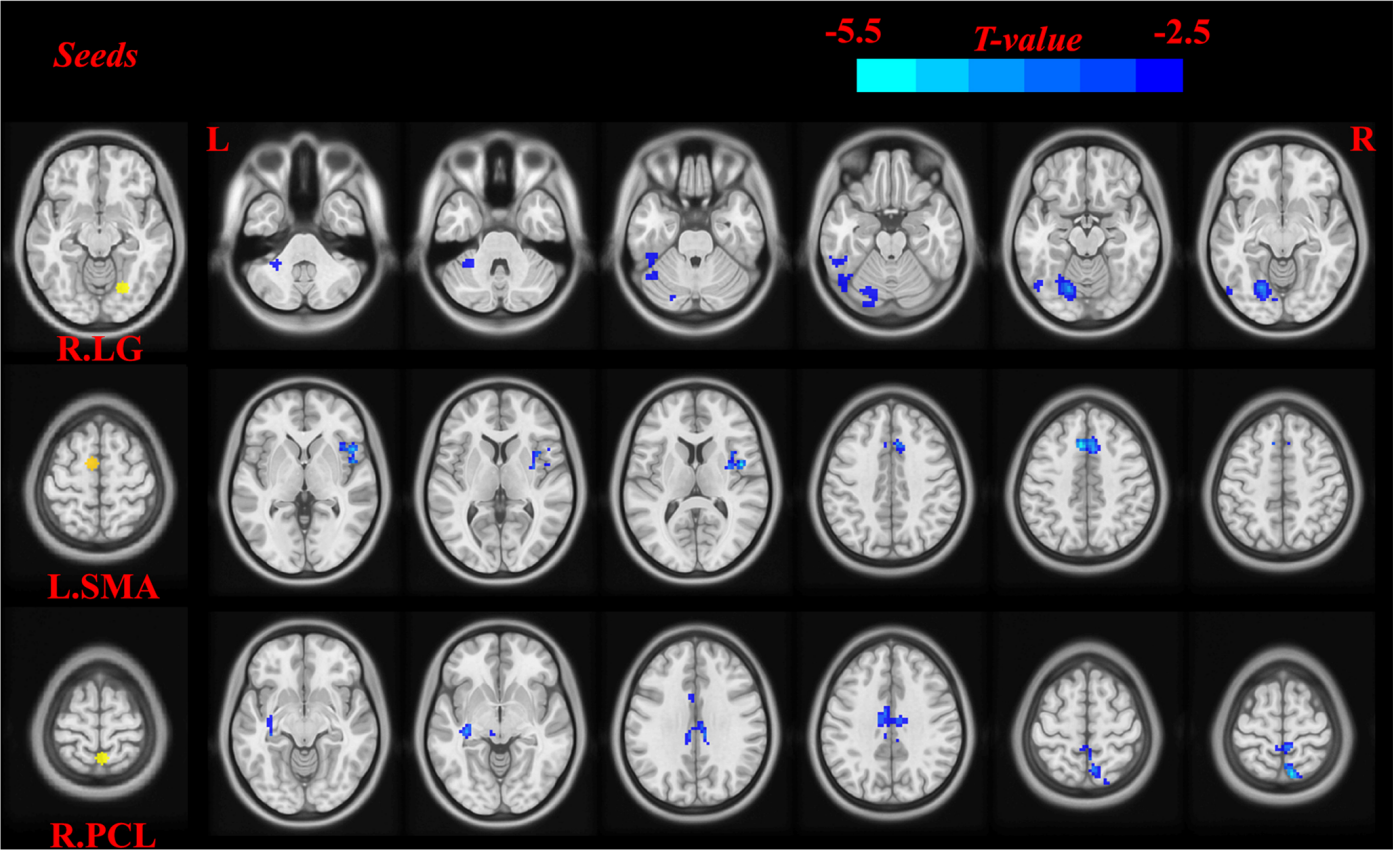
Significantly decreased brain regions in the patients with COPD compared to the NC using seed-based FC (GRF-corrected, voxle level *P*<0.01, cluster level *P*<0.05). Abbreviations: *LG* Lingual gyrus, *SMA* Supplementary motor area, *PCL* Paracentral lobule, *R* Right

We further examined the seed-based FC associated with 3 ROIs (right LG, SMA, and right PCL) that exhibited reduced DC in the patients with COPD compared to NC. The seed-based FC analysis found that COPD patients demonstrated significantly decreased FCs between the those seeds with several brain areas. These brain areas included the left cerebellum anterior lobe, left lingual gyrus, left fusiform gyrus, right insula, right inferior frontal gyrus, limbic lobe, cingulate gyrus, left putamen, left lentiform nucleus, right precuneus, and right paracentral lobule compared to NC (Table 3, Fig. 4).

**Table 3:** Decreased regions of seed-based FC in COPD patients compared to NC

Seeds	Brain regions	BA	Peak MNI			t-value	Cluster (voxels)
			X	Y	Z		
R.LG	Cerebellum Anterior Lobe.L	\	−27	−45	−36	−5.7101	74
	Lingual Gyrus.L	18	−21	−69	−12	−5.8328	130
	Fusiform Gyrus.L	37	−48	−63	−21	−5.0071	76
SMA	Insula/Inferior Frontal Gyrus.R	47	45	0	9	−5.3032	219
	Limbic Lobe/ Anterior Cingulate Gyrus	32	−6	21	45	−5.2845	136
R.PCL	Putamen/Lentiform Nucleus.L	\	−33	−24	−6	−4.3023	250
	Cingulate Gyrus	23	6	−12	33	−4.5513	191
	Precuneus/Paracentral Lobule.R	7	9	−66	66	−5.1566	153

Abbreviations: *COPD* Chronic obstructive pulmonary desease, *NC* Normal controls, *MNI* Montreal neurological institute, *BA* Broadmann area, *R* Right, *L* Left, *LG* Lingual gyrus, *SMA* Supplementary motor area, *PCL* Paracentral lobule, *GRF*-Corrected, voxle level *P*<0.01, cluster level *P*<0.05

### Correlation analysis

**Fig. 5:**
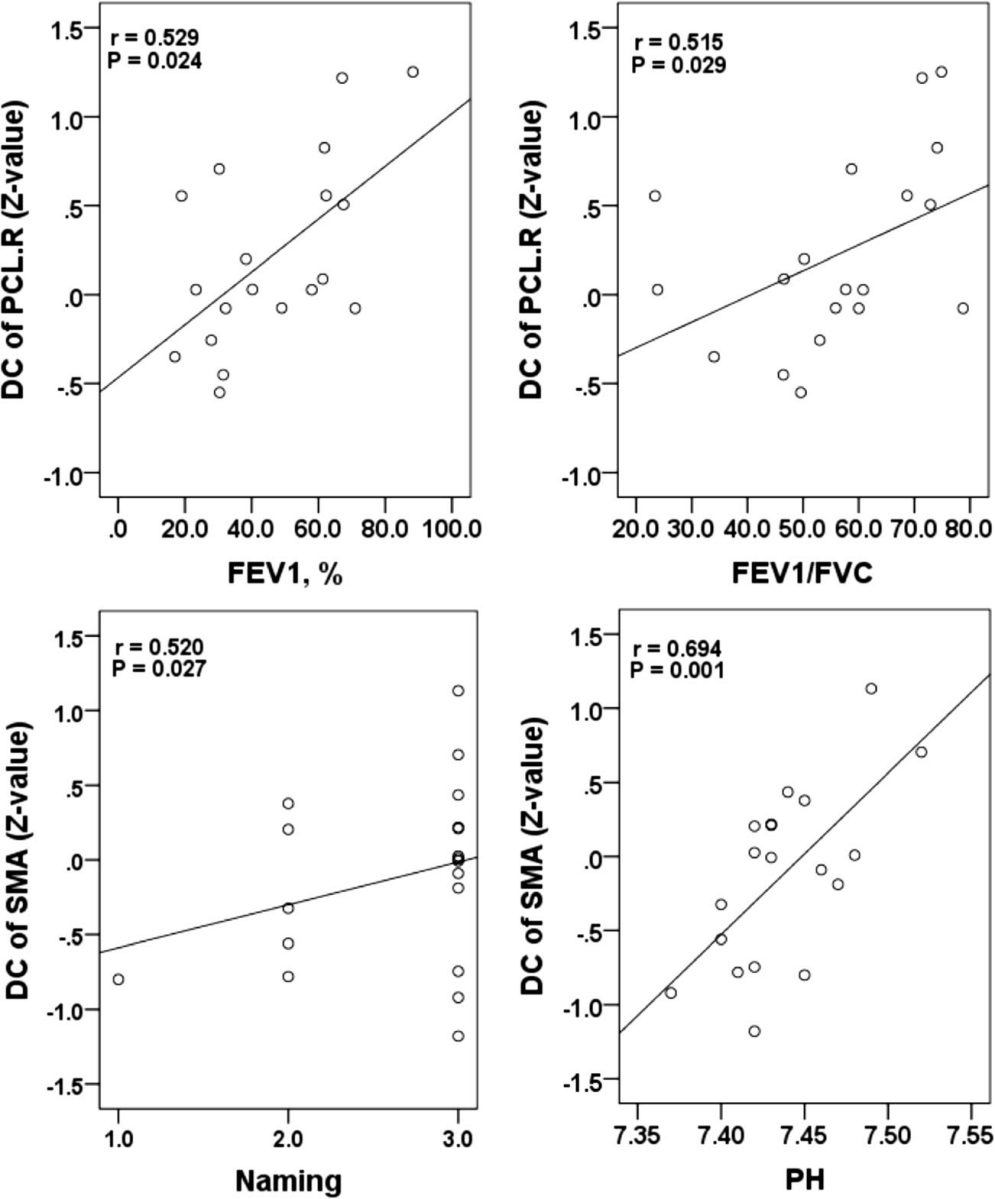
The correlations between altered DC and the clinical variables in patients with stable COPD. Abbreviations: *DC* Degree centrality, *PCL* Paracentral lobule, *SMA* Supplementary motor area, *R* Right, *FEV1* Forced expiratory volume in 1 s, *FVC* Forced expiratory vital capacity, *pH* Power of hydrogen

In the patients with stable COPD, a partial correlation analysis showed that the decreased DC in the right PCL was positively correlated with FEV_1_ (r = 0.529, *P* = 0.024) and FEV1/FVC(r = 0.515, *P* = 0.029); the decreased DC in the SMA was positively correlated with naming (r = 0.520, *P* = 0.027) and pH (r = 0.694, *P* = 0.001) (Fig. 5).

## Discussion

As far as we know, this is the first study to use both DC and FC approaches to investigate the changes in the intrinsic functional architecture of whole brain functional networks and their connectivity related to the possible neurological mechanisms of cognitive impairment in stable patients with COPD. This study displayed remarkably similar higher DC values (functional hubs) in both COPD and NC groups in the specific brain region, which is a large overlap with previous hub mapping studies.(van den Heuvel and Sporns 2013; Achard et al. 2006; Buckner et al. 2009) The present study further demonstrated decreased DC in bilateral SMA and right PCL, which are major hubs in motor networks. Meanwhile, it also showed decreased DC in the right LG in patients with COPD. There is evidence that cigarette smoking may cause white matter integrity damage in patients with COPD,(Dodd et al. 2012) but we found no significant difference in DC after controlling for smoking. In addition, COPD patients showed significantly decreased FCs between those reduced functional hubs with several brain areas. Importantly, decreased DC in the right PCL and SMA revealed significant positive correlations with FEV1, FEV1/FVC, naming, and pH in the stable patients with COPD. These findings suggest that COPD impaired the functional connectivity of the functional network hubs, which was associated with cognitive deficits.

Specifically, our findings showed that the SMA, one of the most important structure of motor notworks, is the primary reduced cortical hub in the brain network architecture affected by COPD. The SMA is involved in the planning, initiation of movements, and prediction of sensorimotor processing.(Makoshi et al. 2011) Previous studies showed reduced cortical thickness, gray matter volume and surface area in the supplement motor area in stable patients with COPD, which was associated with lower arterial oxygen saturation.(Zhang et al. 2012; Chen et al. 2016) These datas indicated that cortical thinning and surface area reduction were crucial morphological characteristics that are partly caused by oxygen desaturation related to COPD.(Chen et al. 2016) A previous study by model-driven methods showed decreased brain neural activation in the left and right primary and premotor cortex, and primary and secondary somatosensory cortex during inspiratory loading. Further voxel-wise functional connectivity analyses found decreased connectivity of the networks linked between the right and left motor cortex and contralateral motor area in COPD patients.(Yu et al. 2016) Consistent with previous studies, the present study found reduced DC in the SMA in stable COPD patients. These results and our findings suggest that the motor cortex is vulnerable. The impairments of cortical thickness, gray matter volume and neuronal activity could contribute to reduced DC in the SMA. A further seed-based FC analysis found that COPD patients demonstrated significantly decreased FCs between the SMA and the right insula, right inferior frontal gyrus, limbic lobe, and anterior cingulate gyrus. The insula has many long-distance connections to the SMA.(Yu et al. 2016) The insula is an integrated sensorimotor area of the limbic system, whose anterior part is activated in response to breath loading, and plays a vital role in dyspnea perception.(Von et al. 2008) The anterior cingulate cortex, together with the anterior insula, could also play a key role in the perception of hypoxemia and may form a neural substrate to control and suppress physiological impulses.(Lerner et al. 2009) Various studies have suggested that COPD patients showed reduced gray matter and cortical thickness in the right insular and anterior cingulate cortex, which was associated with a reduced perception of dyspnea.(Zhang et al. 2013; Chen et al. 2016) A previous study also showed that cognitive dysfunction can be reversed by long-term oxygen therapy in COPD patients.(Negro et al. 2015) The decreased FCs between SMA and these areas contributed to the damaged long-distance connection and structural damage, which is associated with dyspnea and hypoxia. Moreover, a correlation analysis found that decreased DC in the SMA was positively correlated with naming. Therefore, the decreased DC and FC values in the SMA indicated damaged information communication, which may be the behavioral level evidence of cognitive and motor dysfunction.

Our study also showed the significantly decreased DC in the PCL in COPD, which is located in the medial cerebral hemisphere surface, and connects to the medial portions of the precentral and postcentral gyri. The PCL was divided into the anterior two thirds, which is a part of the primary motor area, and the posterior third, reflects the primary somatosensory area.(Jacobs 1992) Similar to OSA, hypoxia and hypercapnia, caused by airflow obstruction, is a major characteristic of COPD. Oxygen deficiency can cause increased blood viscosity and the production of oxygen free radicals, which may lead to neuron damage. Cortical thickness thinning in the anterior paracentral/precentral lobule was found in OSA patients, which is associated with upper airway sensory and motor control.(Macey et al. 2018) A study showed significantly decreased resting-state FC from the right insular to bilateral paracentral lobule in OSA patients, which may further impair sensorimotor function.(Park et al. 2016) A surface-based morphometry assessment has shown cortical thickness in the right PCL, which could be attributed to hypoxia and hypercapnia.(Chen et al. 2016) An electroencephalogram study have found that neurons damaged the processing of incoming mechanical sensory signals in the somatosensory area of the cerebral cortex in severe asthma.(Davenport et al. 2000) Therefore, our findings are consistent with these neuroimaging studies. Meanwhile, the seed-based FC analysis further showed significantly decreased FCs between the PCL and cingulate gyrus, right precuneus, left putamen, left lentiform nucleus, and right PCL in COPD patients. The precuneus and cingulate gyrus are the most important hubs of the default mode network, and significantly abnormal function connection was found in COPD.(Hu et al. 2018) The putamen and lentiform nucleus is part of the basal ganglia, which is to reduce the volume of gray matter.(Zhang et al. 2013) Our study also demonstrated that COPD patients showed that decreased DC in the right PCL was positively correlated with FEV1 and FEV1/FVC. These findings suggest that decreased DC in the PCL may be by a variety of independent factors, including hypoxia**,** hypercapnia, and abnormalities of brain connections.

The LG lies between the posterior portion of the collateral sulcus and the calcarine sulcus, and it has a direct anatomical connection with the visual striate cortices. The LG is a part of the occipitotemporal pathway, which is activated by visuospatial navigation,(Grön et al. 2000) tactile-guided drawing,(Likova 2010) and angle discrimination tasks.(Prvulovic et al. 2002) Zhang et al., found that COPD patients showed significantly reduced ALFF values in the right LG, and it is positively correlated with visual reproduction, indicating it was associated with visual processing.(Zhang et al. 2016) Previous research has also shown the significant reduction of white matter fractional anisotropy in the right side of the LG, which suggests that the damaged integrity of the white matter fiber may lead to reduced neuronal activity.(Zhang et al. 2012) Similar to these results, the current study found that COPD patients showed significantly reduced DC in the LG and lower scores in visuospatial and executive function than to NC, which may supplementally confirm the deficit of LG. Further seed-based FC analysis found that the right LG showed decreased functional connectivity with the contralateral LG and a fusiform gyrus in stable patients with COPD. The fusiform gyrus is commonly involved in visual processing, spatial orientation, and visuospatial processing.(Jahn et al. 2009; Han et al. 2013) One recent voxel-based morphometry study reported significantly lowered gray matter volumes in the bilateral fusiform gyrus, which is susceptible to hypoxic damage.(Wang et al. 2017) These findings indicated that reduced DC and FC in the LG could provide more insight into the underlying mechanisms of visuospatial deficits in COPD patients.

### Limitations

There are several limitations in the current study. First, this research was a cross-sectional, longitudinal study that to explored the DC changes in COPD patients before and after treatment, because drugs may affect brain pathology. Second, the study comprised a relatively small population with stable COPD, so the severity of the disease and acute exacerbation patients cannot be considered, who may display different brain connections. In addition, fMRI based on blood oxygenation level-dependent (BOLD) contrast can indirectly reflects neuronal activity changes by measured cerebral metabolic rate of oxygen changes, which may be affected by hypercapnia and compromised vasculature in COPD. Non-BOLD-fMRI, a useful technology that provides improved neurovascular coupling models, can facilitate the interpretability of fMRI in COPD.(Huber et al. 2017) Finally, DC and FC are undirected connections; therefore large-sample study with new technology, such as eigenvector centrality(Zuo et al. 2012) or granger causality analysis,(Seth et al. 2015) can obtain more information about connectivity patterns that are likely affected by COPD in the future.

## Conclusion

Our findings suggest that there are abnormal intrinsic functional hubs in the right LG, bilateral SAM and right PCL; moreover, ther are connectivity alterations between those hubs with several brain areas in stable patients with COPD. These alterations may be associated with the disruption of visuospatial and executive function deficits, which are partly attributed to hypoxia, and hypercapnia. This study helps us clarify the neurological mechanisms underlying cognitive dysfunction in COPD patients from a whole brain functional connections perspective.

## Electronic supplementary material

ESM 1 (DOC 63 kb)
