# Supplementary material for: Abnormal intrinsic functional hubs and connectivity in stable patients with COPD: a resting-state MRI study
Source: Brain Imaging Behav. 2019 Jun 11;14(2):573–85. doi: 10.1007/s11682-019-00130-7 (PMC7160072; doi:10.1007/s11682-019-00130-7)
Supplement: Supplementary file 1 — (DOC 63 kb) [file 11682_2019_130_MOESM1_ESM.doc]

| **S1** **Table**  Brain areas showed reduced DC between patients with CODP and NC（r0=0.15） | | | | | | | |
| --- | --- | --- | --- | --- | --- | --- | --- |
| Condition | Brain regions | BA | Peak MNI | | | t-value | Cluster (voxels) |
|  |  |  | X | Y | Z |  |  |
| COPD＜NC | Lingual Gyrus.R | 18 | 21 | -69 | -12 | -4.8936 | 107 |
| COPD＜NC | Supplementary Motor Area.L | 6 | -9 | -3 | 60 | -5.0854 | 98 |
| COPD＜NC | Paracentral Lobule.R | 5 | 2 | -54 | 69 | -4.4031 | 73 |
| GRF-corrected，Voxle level P＜0.01, cluster level P＜0.05 | | | | | | | |

| **S2** **Table** Brain areas showed reduced DC between patients with CODP and NC（r0=0.20） | | | | | | | |
| --- | --- | --- | --- | --- | --- | --- | --- |
| Condition | Brain regions | BA | Peak MNI | | | t-value | Cluster (voxels) |
|  |  |  | X | Y | Z |  |  |
| COPD＜NC | Lingual Gyrus.R | 18 | 21 | -69 | -12 | -4.9931 | 147 |
| COPD＜NC | Supplementary Motor Area | 6 | -9 | -3 | 60 | -5.147 | 249 |
| COPD＜NC | Paracentral Lobule.R | 5 | 2 | -54 | 69 | -4.4282 | 114 |
| GRF-corrected，Voxle level P＜0.01, cluster level P＜0.05 | | | | | | | |

| **S3** **Table** Brain areas showed reduced DC between patients with CODP and NC（r0=0.30） | | | | | | | |
| --- | --- | --- | --- | --- | --- | --- | --- |
| Condition | Brain regions | BA | Peak MNI | | | t-value | Cluster (voxels) |
|  |  |  | X | Y | Z |  |  |
| COPD＜NC | Lingual Gyrus.R | 18 | 21 | -69 | -12 | -5.1667 | 114 |
| COPD＜NC | Supplementary Motor Area | 6 | -9 | -3 | 60 | -5.3112 | 160 |
| COPD＜NC | Paracentral Lobule.R | 5 | 2 | -54 | 69 | -4.3048 | 75 |
| GRF-corrected，Voxle level P＜0.01, cluster level P＜0.05 | | | | | | | |

| **S4 Table** Brain areas showed reduced DC between patients with CODP and NC（r0=0.35） | | | | | | | |
| --- | --- | --- | --- | --- | --- | --- | --- |
| Condition | Brain regions | BA | Peak MNI | | | t-value | Cluster (voxels) |
|  |  |  | X | Y | Z |  |  |
| COPD＜NC | Lingual Gyrus.R | 18 | 21 | -69 | -12 | -5.1456 | 151 |
| COPD＜NC | Supplementary Motor Area | 6 | -9 | -3 | 60 | -5.3458 | 158 |
| COPD＜NC | Paracentral Lobule.R | 5 | 3 | -39 | 69 | -4.3821 | 73 |
| GRF-corrected，Voxle level P＜0.01, cluster level P＜0.05 | | | | | | | |

| **S5** **Table** Brain areas showed reduced DC between patients with CODP and NC after control of smoking（r0=0.25） | | | | | | | |
| --- | --- | --- | --- | --- | --- | --- | --- |
| Condition | Brain regions | BA | Peak MNI | | | t-value | Cluster (voxels) |
|  |  |  | X | Y | Z |  |  |
| COPD＜NC | Lingual Gyrus.R | 18 | 21 | -69 | -12 | -4.8075 | 107 |
| COPD＜NC | Supplementary Motor Area.L | 6 | -9 | -3 | 60 | -5.0317 | 96 |
| COPD＜NC | Paracentral Lobule.R | 5 | 2 | -54 | 69 | -4.3609 | 75 |
| GRF-corrected，Voxle level P＜0.01, cluster level P＜0.05 | | | | | | | |
